# Supplementary figures and images for: Single cell RNA-sequencing and RNA-tomography of the avian embryo extending body axis
Source: Front Cell Dev Biol. 2024 May 28;12:1382960. doi: 10.3389/fcell.2024.1382960 (PMC11165230; doi:10.3389/fcell.2024.1382960)

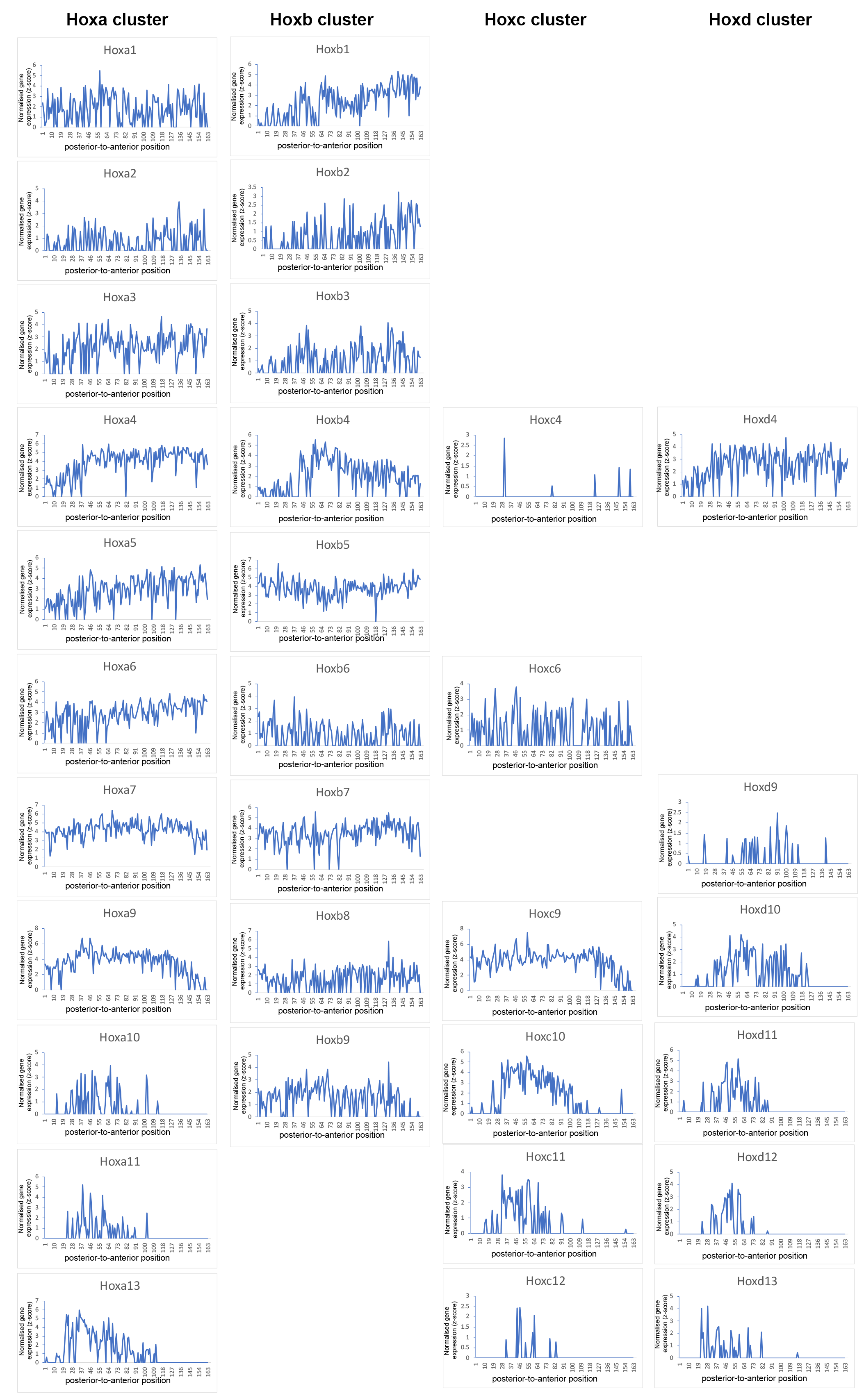

Supplement: Supplementary file 1 [file Image1.TIF]
