# Supplementary material for: Single cell RNA-sequencing and RNA-tomography of the avian embryo extending body axis
Source: Front Cell Dev Biol. 2024 May 28;12:1382960. doi: 10.3389/fcell.2024.1382960 (PMC11165230; doi:10.3389/fcell.2024.1382960)
Supplement: Supplementary file 2 [file DataSheet1.docx]

**Supplementary Figure 1:** **RNA-tomography gene expression profiles of Hox family members.** Line plots show expression along the anterior-posterior embryonic axis for the Hox genes indicated. Note that more posterior members of the clusters have an expression cut-off within the area examined, e.g. *Hoxa10*, *Hoxa11*, *Hoxa13*, *Hoxc10*, *Hoxc11*, *Hoxc12*, *Hoxd9*, *Hoxd10*, *Hoxd11*, *Hoxd12* and *Hoxd13*. These profiles are in agreement with the bulk RNA-sequencing of paraxial mesoderm tissues published in Mok (2021).

**Supplementary Table 1: List of genes in k-means cluster 3 grouped for the indicated gene ontology (GO) terms**

| GO term | Genes included |
| --- | --- |
| neurogenesis | Ngfr, Gabra2, Serpinf1, Ckb, Nes, Aldh1a2, Rarb, Hoxb1, Rfx4, Ntn1, Ptch1, Sema3g, Col2a1, Cdh2, Slit1, Gfra1, Nfasc, Prrx1, Pax6, Meis2, Postn, Sfrp2, Epha7, Dab1, Pax7, Cd38, Foxp2, Plxna4, Ptn, Lrig1, Nr2f2, Vegfd, Fgfr1, Gnao1, Cadm1, Gpc1, Lrrn1, Prtg, Wnt4, Cdon, Sema3d, Nbl1, Nkx6-2, Reln, Fat4, Hes5, Robo2, Jam2, Musk, Ctnna2, Plppr1, Ptprz1, Slc8a3, Ntn3 |
| pattern specification | Meox1, Aldh1a2, Tbx5, Hoxb1, Rfx4, Ptch1, Fst, Pax6, Meis2, Sfrp2, Pax7, Lfng, Nr2f2, Tbx22, Fgfr1, Cdon, Hoxa5, Nbl1, Reln, Hoxa6, Hes5, Nkx3-2, Acvr2b, Tcf15 |
